# Elaboration and Characterization of Novel Kombucha Drinks Based on Truffles (*Tuber melanosporum* and *Tuber aestivum*) with Interesting Aromatic and Compositional Profiles

**DOI:** 10.3390/foods13132162

**Published:** 2024-07-08

**Authors:** Diego Morales, Laura de la Fuente-Nieto, Pedro Marco, Eva Tejedor-Calvo

**Affiliations:** 1 Departmental Section of Galenic Pharmacy and Food Technology, Veterinary Faculty, Complutense University of Madrid, 28040 Madrid, Spain; ldelafue@ucm.es; 2 Department of Plant Science, Agrifood Research and Technology Centre of Aragón (CITA), Avenida Montañana 930, 50059 Zaragoza, Spain; pmarcomo@cita-aragon.es; 3 Laboratory for Flavor Analysis and Enology (LAAE), Department of Analytical Chemistry, Faculty of Sciences, Instituto Agroalimentario de Aragón (IA2), University of Zaragoza, 50009 Zaragoza, Spain

**Keywords:** kombucha, truffle, fermentation, aroma, SCOBY, phenolic compounds

## Abstract

The organoleptic and bioactive properties of truffles place these fungi as interesting materials for use in the of design functional foods based on fruiting bodies outside commercial standards. Moreover, kombucha beverages have become more popular in the Western world, leading to novel drinks using alternative substrates instead of tea leaves. In this work, two truffle species (*Tuber melanosporum*, TMEL; *Tuber aestivum*, TAES) and three different symbiotic consortia of bacteria and yeasts (SCOBYs: SC1, SC2, and SC3) were tested. Fermentation (21 days) was monitored in terms of physicochemical (pH, viscosity), biochemical (total carbohydrates, alcohol, soluble proteins, phenolic compounds), and sensory attributes (volatile organic compounds, VOCs). The obtained pH ranges were adequate, alcohol levels were undetectable or very low, and sugar content was lower than in traditional kombuchas or other beverages. In most cases, the usual bottling time could be applied (7–10 days), although longer fermentations are recommended (14 days) to reach higher protein and phenolic compounds contents. Truffle kombuchas produced up to 51 volatile organic compounds (alcohols, acids, esters, ketones, and aldehydes, among others), with TMEL showing a more complex profile than TAES. During the first week, acidic compound production was observed, especially acetic acid. Similar behavior in the VOC profile was reported with different SCOBYs.

## 1. Introduction

Truffles are valued worldwide for their gourmet organoleptic properties. Nowadays, truffles’ commercial categorization is followed by a non-mandatory standard for the marketing and commercial quality control of truffles (Unece Standard FFV-53) [1]. Since this recommendation classifies truffles morphologically and by weight, many truffle fruiting bodies do not fit in any of the categories, but they still contain aromatic compounds [2]. 

Among *Tuber* truffle species, the two most cultivated are *Tuber melanosporum* (TMEL), also known as black truffle, and *Tuber aestivum* (TAES) or summer truffle. Both are originally from Mediterranean countries (Spain, France, and Italy), but in the case of the black truffle its cultivation has been extended over the world [3]. These sorts of truffles produce bioactive compounds and enriched extracts obtained from these species have been demonstrated to exert antioxidant, antiproliferative, and antidiabetic properties, among others [4,5]. The truffle samples or their extracts might be used as functional ingredients to develop or improve food products, and therefore, the mentioned fruiting bodies that cannot be commercially used can and must be revalorized.

Recently, the popularity of kombucha in the Western world has been increased. This drink originated in northeastern China around 200 B.C., being traditionally prepared with tea leaves and sugar that were fermented by a consortium of bacteria and yeasts known as ‘SCOBYs’ (symbiotic consortium of bacteria and yeasts) [6]. During the last years, alternative substrates have been tried to replace tea partially or totally, leading to innovative kombuchas with interesting aromatic and compositional properties based on fruits, herbs, etc., [7,8,9,10]. However, just two works have targeted fungal species (mushrooms) as raw materials [11,12] and no truffle kombuchas have been tested yet.

Therefore, the aim of this work was to design and characterize novel kombucha beverages with black and summer truffles using three SCOBYs (SCs) with different microbial composition. For that, physicochemical (pH, viscosity), biochemical (total carbohydrates, alcohol, proteins, phenolic compounds), and sensory (volatile organic compounds) parameters were monitored during 21 days of fermentation.

## 2. Materials and Methods

### 2.1. Biological Material

*Tuber melanosporum* (Vittad.) and *Tuber aestivum* ascocarps were collected in Zaragoza province (Aragon, Eastern Spain) throughout the harvesting season 2022/2023. Then, truffles (20 units/species) were authenticated by the morphological features of the spores [13,14], selected, and processed under refrigeration following Rivera et al.’s (2011) indications [15]. Fresh truffles were freeze-dried (LyoBeta 15 lyophilizer, Telstar, Madrid, Spain), ground, and sieved to obtain a particle size lower than 0.5 mm and stored in darkness at −20 °C until further use. Three different SCs were utilized: SC1, kindly provided by a local producer; SC2, purchased in “Oh My Kefir!”; and SC3, previously prepared in the laboratory following the procedure described by Tejedor-Calvo and Morales (2023) [16]. Briefly, tap water (3 L) was heated up to 80 °C prior to the addition of powdered dried green tea leaves (8 g/L) (Hornimans, London, UK) and white cane sugar (80 g/L) (Acor, Valladolid, Spain). After vigorous stirring, the homogenized mixture was cooled down to room temperature (RT). Then, non-pasteurized commercial kombucha (275 mL) (Kombutxa, Mataró, Spain) was added to the mixture; it was then transferred to a glass vessel that was kept opened but covered with filter paper. The vessel was stored in darkness at RT for 3 weeks.

### 2.2. Reagents

Methanol (HPLC) was obtained from LAB-SCAN (Gliwice, Polland) and sodium carbonate (Na_2_CO_3_) and sulfuric acid (H_2_SO_4_) from Panreac (Barcelona, Spain). Bovine serum albumin (BSA), HCl (37%), phenol, Folin Ciocalteu’s phenol reagent, D-glucose, and gallic acid were purchased from Sigma-Adrich Quimica (Madrid, Spain), as well as the n-alkane series and standards for MS identification (all standards of purity > 95%). The Bradford reagent was acquired from Bio-Rad (Hercules, CA, USA). 

### 2.3. Kombucha Elaboration with SCOBYs

The truffle kombuchas were prepared with the different SCs (in duplicate) according to the protocol described by Tejedor-Calvo and Morales (2023) [16], with slight modifications. Briefly, freeze-dried truffle powder (12 g/L) was added to hot tap water (80 °C) together with white cane sugar (70 g/L). After vigorous stirring, the homogenized suspension was cooled down to RT before including SC1, SC2, or SC3 (40 g/L) and 10% *v*/*v* of the medium (‘old kombucha’), in which the SCOBY was immersed. The vessels with the kombuchas were kept opened, covered with filter paper, and stored in darkness at RT for 21 days. 

### 2.4. Physicochemical Measurements

An HI 4211 Hanna pH meter (Hanna Instruments, Eibar, Spain) was used to measure the pH, and a digital rotational viscosimeter Visco Star R (Fungilab S.A., Sant Feliu de Llobregat, Spain) was used to determine the viscosity of all the kombuchas at different fermentation times (day 0, 3, 7, 10, 14, 17, and 21).

### 2.5. Biochemical Measurements

The total carbohydrate content was determined using the phenol–sulfuric method, as detailed by Morales et al. (2023) [17]. D-Glucose was used as a standard for quantification. The ethanol content was measured using a manual refractometer, ALL001 (Allmeter, London, UK), and results were expressed as % (*v*/*v*). The total soluble protein concentration was determined using the Bradford method reagents (Sigma-Aldrich, Madrid, Spain) according to the instruction manual, using bovine serum albumin as the standard for quantification. The total phenol concentration was calculated by the Folin–Ciocalteu method following the procedure described by Ramírez-Anguiano et al. (2007) [18], using gallic acid as the standard for quantification. All measurements were studied in the kombuchas at different fermentation times (day 0, 3, 7, 10, 14, 17, and 21).

### 2.6. Volatile Organic Compound (VOC) Analysis by SPME-GC-MS

The methodological approach was carried out according to Tejedor-Calvo et al. (2021) [2]. In order to extract the volatile compounds, a solid phase microextraction (SPME) was used. For that, a fused silica fiber coated with a 50/30 mm layer of divinylbenzene/carboxen/polydimethylsiloxane from Supelco (Barcelona, Spain) was chosen. The samples (2 mL of liquid kombucha) were placed in a 20 mL glass vial closed with a septum. The vial was conditioned at 50 °C for 10 min followed by 20 min of fiber exposition to the headspace of the vial. 

The VOC profiles of the different samples were analyzed by static GC–MS using a gas chromatograph Agilent 6890 N (Termoquest, Milan, Italy) coupled with a mass spectrometer detector. This instrument was equipped with a capillary column HP-5MS (Agilent Technologies, Santa Clara, CA, USA) of 30 m, 0.32 mm i.d., 0.25 μm film thickness, and a flow of 1 mL/min with helium as a carrier gas. The oven temperature was 45 °C held for 2 min, 45–200 °C at a rate of 4 °C/min, and finally, to 225 °C at 10 °C/min and held for 5 min [19]. The MS used the electron impact mode with an ionization potential of 70 eV and an ion source temperature of 200 °C. The interface temperature was 220 °C. The MS scanning was recorded in full scan mode (35–250 *m*/*z*). The TurboMass software (version 6.1) (Perkin Elmer, Shelton, CT, USA) was used for controlling the GC–MS system.

The peak identification of the VOCs was achieved by comparison of the mass spectra with mass spectral data from the NIST MS Search Program (library version 2.0), and by comparison of previously reported retention indexes (RIs) with those calculated using an n-alkane series (C6–C20) under the same analysis conditions. Semi-quantification was achieved by integrating the area of the sum of ion characteristics of each compound and normalization by calculating the relative percentage. This allowed comparison of each eluted compound between samples.

### 2.7. Statistical Analyses

Differences were evaluated at a 95% confidence level (*p* ≤ 0.05) using a one-way analysis of variance (ANOVA) followed by Tukey’s multiple comparison test. Statistical analysis was performed using GraphPad Prism version 9.5.1 (GraphPad Software, San Diego, CA, USA). The VOC data were analyzed with the MetaboAnalyst 5.0 software (http://www.metaboanalyst.ca, accessed on 6 February 2024). Missing values were replaced with the smallest values with which a feature could still be detected. Also, sum normalization and autoscaling were performed. Afterwards, plots of principal component analysis (PCA) or partial least squares discriminant analysis (PLS-DA) were computed to estimate variance, sample distribution, and homologies between sample groups. The relevant marker substances were selected by carrying out analysis of variance (ANOVA) as well as using false discovery rates (FDRs) [20].

## 3. Results and Discussion

### 3.1. Evolution of pH and Viscosity in Truffle Kombuchas during Fermentation

**Figure 1:**
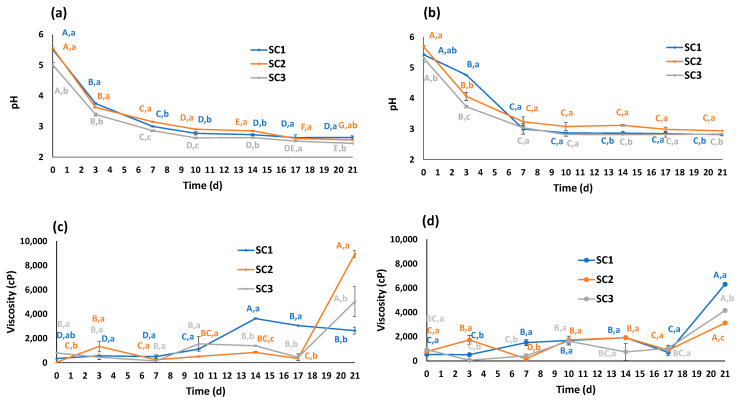
Evolution of pH values and viscosity (cP) during fermentation (21 days) in *Tuber melanosporum* (**a**,**c**) and *Tuber aestivum* (**b**,**d**) kombuchas with SCOBYs (SCs) 1 (blue), 2 (orange), and 3 (gray). Different letters denote significant differences for the same SC at different fermentation times (A–G) and for different SCs at the same fermentation time (a–c) (one-way ANOVA, Tukey’s test, *p* ≤ 0.05).

The initial pH values for both truffle kombuchas (Figure 1a,b) were in a similar range for all the tested SCs, between 5.01 and 5.69, being slightly lower in the case of kombuchas fermented with SC3.

In all cases, these starting levels were higher than those recommended for avoiding the growth of undesirable microorganisms (≤4.2) [21]. Thus, although no mold-like fungi presence was observed in any of the fermentation processes (these fungal species are the most usual contamination in kombucha cultures), the addition of greater volumes of ‘old kombucha’ is suggested for further preparations [22]. A rapid decrease in pH values was noticed during the first week, particularly between days 0 and 2, registering reduction rates between 32 and 36% and 12 and 29% for TMEL and TAES, respectively. After this drastic decline, the decrease was significantly less during the last 2 weeks, avoiding the excessive production of acetic acid. As a matter of fact, after 21 days, the pH range was 2.45–2.94, and only TMEL kombucha fermented with SC3 showed a pH below the safe limit (2.5), linked to too high acetic acid concentrations [21]. Moreover, since conventional kombuchas are bottled after 7–10 days of fermentation, this extremely low level that only occurred in prolonged processes would be avoided. As previously commented, SC3 gave rise to more acidic drinks, possibly due to the specific composition of the consortia, with fewer counts of yeasts and higher levels of lactic acid bacteria (LAB) when compared to the other SCs [16]. The potent pH-decreasing activity of LAB and LAB-rich SCs was previously observed in tea kombuchas [23,24].

Similar tendencies were noticed in kombuchas prepared with the same SCs but using fruits as substrates (cherry, persimmon, pomegranate) [17]. However, since the initial pH was significantly lower (3.3–3.4), these beverages did not show the severe reduction reported at day 2 for truffle kombuchas. The increase in medium acidity was totally expected due to the activity of acetic acid bacteria (AAB) that generated acetic, gluconic, and glucuronic acid, and the release of truffle organic acids from the fungal matrix to the liquid media [25,26].

Regarding the viscosity evolution during the studied fermentation time (Figure 1c,d), all samples showed initial low values, with the lowest one in TMEL samples fermented with SC2 (38.5 cP), approximately in the range of those reported in traditional tea kombucha (68.0 cP) in previous works [27]. Although a time-dependent increase was expected, based on the growth of SC microorganisms and the production of cellulose, the most remarkable peak was registered after 3 weeks for both truffles and SCs (except for TMEL fermented with SC1). At this moment, the viscosity levels showed a wide range (2640.0–8945.0 cP) depending on the truffle–SC combination. Just a few available data addressing kombucha viscosities can be found in the scientific literature: Watawana et al. (2016) fermented coconut water with a kombucha consortium for 1 week, observing a significant increase, leading to viscosity levels of 440 cP at day 7, being in the same range as the present study [28]. Tomar (2023) obtained different results for grape, mulberry, and rosehip kombuchas, since the values were lower and dropped during their 21-day fermentation process, being reduced from 56 to 34 cP, for instance, in the case of rosehip fruits [27]. Recently, it has been observed that the addition of kombucha bacterial cellulose can improve the rheological properties of food products such as wheat dough [29].

### 3.2. Evolution of Chemical Composition in Truffle Kombuchas during Fermentation

#### 3.2.1. Evolution of Total Carbohydrate Content (TCC)

**Figure 2:**
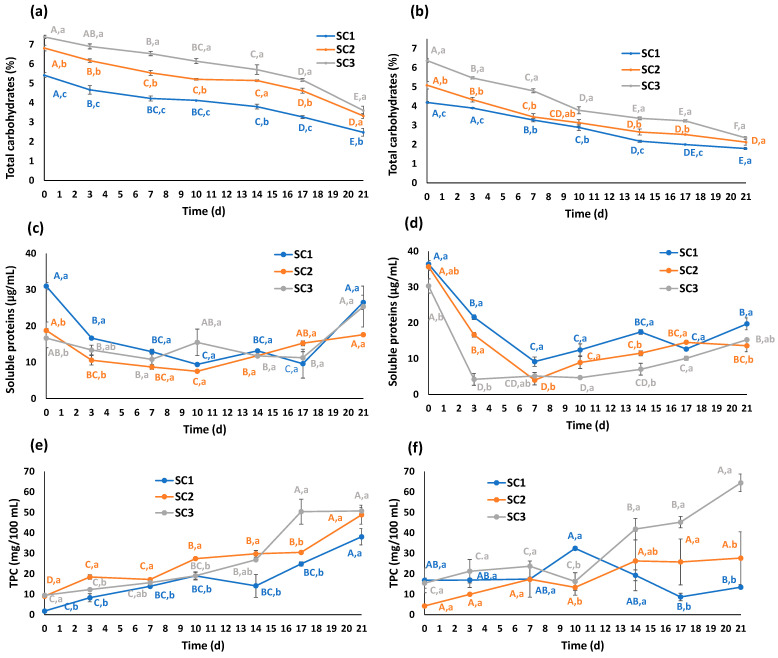
Evolution of total carbohydrate content (%), soluble proteins (µg/mL), and total phenolic compounds (TPCs) (mg/100 mL) during fermentation (21 days) in *Tuber melanosporum* (**a**,**c**,**e**) and *Tuber aestivum* (**b**,**d**,**f**) kombuchas with SCOBYs (SCs) 1 (blue), 2 (orange), and 3 (gray). Different letters denote significant differences for the same SC at different fermentation times (A–F) and for different SCs at the same fermentation time (a–c) (one-way ANOVA, Tukey’s test, *p* ≤ 0.05).

Prior to the start of the fermentative process, the TCC of the samples significantly differed because of the utilized truffle species and because of the applied SC (Figure 2a,b).

First, TMEL kombuchas showed higher TCC than TAES kombuchas (more than 1% higher for the same SC), attributable to the dissimilar compositions of black and summer truffles [30,31]. Moreover, the unequal supply of carbohydrates from SC displayed the same tendency regardless of the truffle species: SC3 kombuchas showed the highest levels, followed by SC2, and finally, SC1, suggesting different TCCs in the SCs and their old kombuchas, since the microbial but also chemical compositions of these consortia were varied [16]. As expected, all truffle kombuchas experienced a remarkable decrease in their TCC due to the carbohydrate utilization by SC microorganisms, reducing the initial values (4.2–7.4%) to significantly lower levels after 1 week (3.1–6.5%) and even lower ones when the whole process was finished after 21 days (1.8–3.6%). Attending to the consumption rates, the results suggested that the studied SCs, particularly SC2 and SC3, were more active when the substrate was TAES, improving the reduction in TCC after 7 (32% for TAES and 19% for TMEL with SC2; 25% for TAES and 12% for TMEL with SC3) and 21 days (58% for TAES and 51% for TMEL with SC2; 63% for TAES and 51% for TMEL with SC3), indicating that TAES carbohydrates were more easily usable for the microorganisms that compose these SCs.

Similar behaviors were observed in a previous work based on mushroom kombuchas, specifically prepared with shiitake (*Lentinula edodes*) and yunzhi (*Trametes versicolor*) mushrooms, with an an even stronger decrease in kombucha sugars noted due to microbial activity. In this case, sucrose was reduced in 1 week from 6.8 and 6.5% to 0.6 and 1.0% in shiitake and yunzhi kombuchas, respectively. However, these values were not further diminished after 11 days. Moreover, glucose and fructose were totally consumed after 7–11 days, starting from ranges of 3.1–3.3% and 1.2–1.5%, respectively [11]. This more active consumption can be attributed to the differences in the substrates (although shiitake and yunzhi are also fungal sources, they are not truffles, and their composition is significantly different) [32] or the microbial consortia [33], that led to dissimilar rates when compared to, for instance, traditional kombuchas prepared with green or black tea, where the initial sucrose content (5%) was reduced after 10 days to 2% and 3.5%, respectively [21]. As a matter of fact, the SC composition might be crucial for carbohydrate fermentation, particularly attending to the counts of yeasts and the presence of certain species and strains with high or low fermentation rates [34]. 

Considering that the bottling time is usually between 7 and 10 days, the TCC ranges registered in the current work were 4.1–6.5% and 2.9–4.8% for TMEL and TAES kombuchas, respectively. From a nutritional and functional perspective, the obtained values were lower than those reported for traditional tea kombuchas prepared through the same methodology (9.5% at day 7) [17] and similar or slightly lower (particularly for TAES) when compared to tea infusions (5–6%) [35]. Moreover, the sugar content in other drinks such as fruit juices or carbonated drinks is significantly higher (9–10%) [36]. Therefore, although a high amount of sugar was added in the kombucha recipes, the obtained insights place truffle kombuchas, particularly those prepared with TAES, as a healthy alternative with lower sugar content.

#### 3.2.2. Evolution of Alcohol Content

One of the main disadvantages related to the fermentative activity of kombucha SCs is the ethanol production during the process. With the aim of obtaining functional beverages with potentially healthy properties, the final products must be alcohol-free, since drinks with >1.2% alcohol volume must be labeled as alcoholic beverages and they are excluded from nutritional or health claims [37]. Attending to the obtained values in truffle kombuchas (Table 1), this 1.2% limit was only surpassed by TMEL kombuchas fermented with SC2 (1.5% at day 7) and SC3 (1.6 and 1.5% at day 7 and day 10). 

These rises might be explained by two factors: (1) TMEL kombuchas showed higher initial TCC than TAES ones, and (2) SC2/SC3 were the most active consortia in terms of TCC reduction rates. Thus, TAES kombuchas did not reach high alcohol contents (≤0.7%), as well as those fermented by SC1, regardless of the truffle species (≤0.25%). In all cases, the alcohol content evolution followed similar tendencies, being undetectable at the beginning of the fermentation, reaching the highest peaks at days 7–10, and rapidly decreasing to undetectable values again because of the action of AAB. This up and down movement in alcohol levels was also observed in traditional tea kombuchas. For instance, Villarreal-Soto et al. (2019) registered significantly higher alcoholic degrees, up to 4.1%, after 11 days in black tea kombucha, being able to be reduced just to 1.4% after 21 days [38]. These higher contents were also registered for green tea kombucha prepared with the same SCs as in the current work, exceeding the 1.2% limit from day 2 to day 11 (1.7–2.6%), but eliminating all the ethanol after 21 days [17]. The mentioned research also tested fruits as alternative substrates, showing different values depending on the fruit, highlighting higher values for cherry kombuchas and very low values for strawberry or pomegranate ones [17].

In the case of kombuchas that were prepared with more similar materials such as mushrooms, interesting results can be found in the literature: shiitake kombuchas experienced a noticeable increase in ethanol content, from undetectable values to 4.3% after 3 days followed by a slight decrease to 3.5% at day 11. When the same SC was used in yunzhi kombuchas, the rise was more gradual (registering the maximum peak at day 2, 3.1%), and the final levels after 11 days were lower (0.4%). In both beverages, the results suggested that longer times must be required to allow AAB to completely oxidize the produced ethanol [11].

#### 3.2.3. Evolution of Soluble Protein Content

Although in traditional and commercial kombuchas the soluble protein content is not high in comparison with other macronutrients (approx. 3 µg/mL) [39], the soluble protein content was evaluated in truffle kombuchas (Figure 2c,d), showing significantly greater values, up to 31.0 and 36.4 µg/mL for the TMEL and TAES beverages, respectively. Some differences could be observed at the starting point: TAES provided more soluble proteins than TMEL since these truffles showed different compositions [30,31]. Moreover, kombuchas including SC1 showed higher protein content than SC2 and SC3, with these differences being significantly marked for TMEL drinks. All samples experienced an initial decrease until day 7 (or day 10 for TMEL fermented with SC1 and SC2), and after this fall they reached values that were similar or higher than the initial ones in the case of TMEL kombuchas but lower for TAES. These fluctuations can be explained by varied phenomena. First, the reduction in soluble protein content might be due to the acidification of the media, since low pH can lead to protein denaturation and pH changes can also provoke precipitation of truffle or microbial proteins. Moreover, degradation of these molecules could have occurred because of microbial activity and the long time that they stayed in liquid solutions. On the other hand, the later increase in protein levels can be justified by the growth of microbial biomass and the consequent increased production of microbial proteins [16,22,40].

Regarding the described behavior, although in truffle kombuchas the initial reduction was followed by a subsequent rise, other tendencies can also be observed when other conditions are settled. For instance, Jayabalan et al. (2007, 2014) reported an inverse order in black tea kombucha: protein content was increased until day 12 and then diminished until the end of the fermentation (18 days). They also observed a decrease in yeast and bacteria counts, suggesting an expected reduction in yeast and bacterial extracellular protein production [22,40]. Therefore, the utilized consortia constitute a key factor, as well as the selected substrate: as a matter of fact, kombuchas prepared with a different substrate, strawberry tree fruits, displayed a constant decreasing tendency for the whole fermentation process (21 days), suggesting that the microbial growth was not enough to counteract the degradation events [16]. No previous reports could be found determining the protein content in kombuchas prepared with truffles or mushrooms, so comparisons with similar raw materials could not be carried out.

#### 3.2.4. Evolution of Total Phenolics Content (TPC)

The initial TPC that was determined in the kombucha samples (Figure 2e,f) varied depending on the truffle species and also the SC, since these consortia were included together with the old kombucha, that provided different TPC to the preparations [16,17]. These starting values ranged between 1.7 and 16.8 mg/100 mL, being the lowest levels that were registered in the whole fermentation process, showing a clear upward trend, except for the decreases that were reported for TAES kombucha fermented with SC1 after day 10. As was described for protein content evolution, different events may affect TPC during fermentation. In this regard, the low pH and the intense microbial activity, together with the enzymatic release of phenolic molecules (including acid hydrolysis, depolymerization, etc.), are linked to the increase in small monomers and low-molecular-weight species that significantly enhance the TPC counts. On the contrary, this acidic environment, and the microbial degradation and polymerization of small phenols, might explain the reduction events that took place also in some previously tested kombuchas [17,41], as well as in some of the truffle-based ones. As mentioned for other constituents such as carbohydrates, the specific composition of the SCs is crucial. It has been demonstrated that yeasts, LAB, and AAB are able to modify (poly)phenolic composition and profiles in food fermentations and, in the opposite sense, phenolics can affect microbial populations [42,43,44]. 

The highest TPC that was observed in this work was for the AES kombucha fermented with SC3, reaching 64.4 mg phenolics/100 mL. This value was significantly higher than those reported for mushroom kombuchas, up to 19.0, 24.5, and 33.0 mg/100 mL for shiitake, reishi, and yunzhi kombuchas, respectively [11,12]. The mentioned content was even higher than the values that were reported for traditional green tea kombuchas (27.0 mg/100 mL) or alternative ones prepared with substrates such as strawberry tree fruits (16.3 mg/100 mL) but with the same or very similar SCs [16,17]. Therefore, truffles, particularly TAES, constitute a very promising ingredient to prepare phenolic-rich fermented beverages.

### 3.3. Evolution of VOCs in Truffle Kombuchas during Fermentation

A total of 51 VOCs (18 esters, 13 acids, 8 alcohols, 2 aldehydes, 4 alkanes, 4 ketones, and 2 hydrocarbon aromatics) were detected in kombucha beverages throughout the 21-day fermentation process (Table 2). 

Black truffle kombuchas at day 0 mainly contained hexane (49.7–66.5% depending on the SCOBY used), followed by 2-propanone (5.0–6.9%), and 2-methyl-butanoic acid (2.9–10.0%). The TAES kombuchas showed similar profiles but with higher contents of hexane (60.0–75.7%) and 2-propanone (5.8–16.6%) (Table S1). The differences noticed between truffle species kombuchas were more related to the truffle composition than the SOCBY used to prepare the kombucha. The same SCOBYs were used to make strawberry tree fruit kombucha, and only 20 VOCs were detected [16]. The increase in the number of compounds might be due to the use of truffles, since they are quite aromatic and more than 200 VOCs have been reported [2,45,46]. 

During the fermentation process, the VOC profile changed in both truffle species kombuchas. After 7 days, the contents of ethyl acetate and acetic acid raised up to 46.3 and 42.5% in *T. melanosporum* and *T. aestivum* kombuchas, respectively (Table S1). Other acids and esters such as pentanoic acid, ethyl nonanoate, and ethyl octanoate were also increased. Acetic acid was reported as the main acid after 21 days of the fermentation process. It achieved ranges from 53.2–63.5% in *T. melanosporum* kombucha and 47.9–66.3% in *T. aestivum* kombucha. During kombucha preparation, the bacteria and yeast cooperate and compete, whereas different acids, such as acetic acid, gluconic acid, and glucuronic acid, are produced [9]. Indeed, some of the acids detected (acetic acid, pentanoic acid, hexanoic acid, heptanoic acid, octanoic acid, and decanoic acid) increased after 7 days of fermentation in both truffle kombuchas. The acid compound production matched the strong decrease in pH values (first 7 days). In addition, the compound acetoin, a ketone, also increased in both types of kombucha during fermentation. This increase had similar behavior when different SCOBYs were applied in the truffle kombucha production. Acetoin is a compound with a buttery flavor, and it is produced by the decarboxylation of alpha-acetolactate, a common precursor in the biosynthesis of branched-chain amino acids [47]. It has been reported that this molecule is excreted to prevent over-acidification [47], therefore its increase after 21 days might be due to modulating the acid production, especially acetic acid.

Ethyl esters have been considered as relevant odorants in many foods due to their fresh and fruity odors. Most of them are the result of the esterification of fatty acids and alcohols produced by yeasts during alcoholic fermentation by lactic and acetic bacteria and constitute one of the largest and most important groups of compounds involved in the flavor of fermented beverages [48]. Among ethyl esters, ethyl acetate, characterized by a pineapple odor, reached 16% in *T. melanosporum* kombucha, and 24.8% in *T. aestivum* kombucha at day 7. Others, such as ethyl octanoate (fruit aroma) and ethyl nonanoate (no odor), achieved levels around 9.4% and 25.0% in *T. melanosporum* kombucha, while ethyl hexanoate (apple peel aroma), ethyl heptanoate (fruit aroma), ethyl decanoate (grape aroma), and ethyl laurate (leaf aroma) were found at less than 2%. The alcohol decline at day 21, mainly due to ethanol, 3-methyl-1-butanol, and 2-methyl-1-butanol, may be attributed to the possible esterification of these alcohols with the available acids in the unfermented sample, contributing to the production of highly aromatic fermented products. Also, ketones are involved in the esterification process. In this sense, the 2-propanone decrease from 5.5–6.9% to less than 0.5% at day 7 in TMEL, and 5.8–16.6% to 1% at 7 days and less than 0.2% at 21 days in TAES samples, might be explained by this chemical reaction to produce acids. 

**Figure 3:**
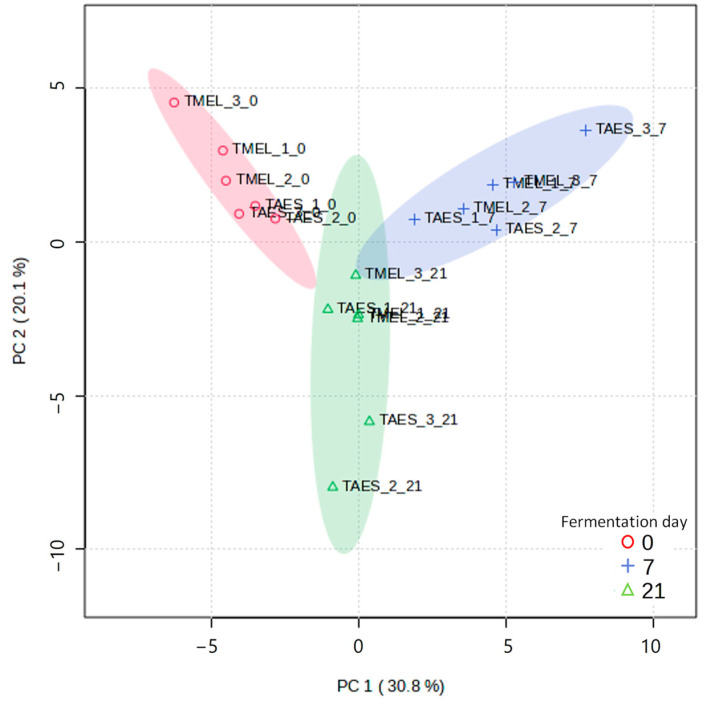
PCA score plot with all volatile organic compounds detected in truffle kombucha fermented during 21 days. In the sample name, TMEL: *Tuber melanosporum*, TAES: *Tuber aestivum*; 1, 2, and 3 correspond to the SCOBY and 0, 7, and 21 to the fermentation day.

A PCA was used to explore possible correlations between VOCs and fermentation day (Figure 3). 

The group distribution in the PCA was clear with no overlays, even including both of the truffle species. The PCA analysis explained 50.9% of the data variability with the two first components. The compounds that showed the more positive loadings within the first PCA component were 4-heptanol,2,6-dimehtyl and phenethyl acetate, whereas those showing the more negative loadings were undecane and 2-propanone. The second component separated the compounds, with isoamyl acetate corresponding with the more negative loading and 2-methyl-1-butanol the more positive loading. When the PCA analysis was carried out regarding the truffle species separately, the PCA explained 66.1 of the data variability for *T. aestivum* samples and 71.3% for *T. melanosporum* samples. Both PCAs showed a clustering depending on the fermentation day. However, *T. melanosporum* kombucha showed a better group differentiation, probably because this truffle species has been characterized as more aromatic than the summer truffle [2]. Also, it is possible that the fermentation process with this species had more effect on the aromatic profile. The outcome of a PLS-DA calculation along with the corresponding leave-one-out cross validation (LOOCV) supported these results. The Q^2^-value was 0.92 for one component and 0.96 for two components, demonstrating a clear separation of the three sample groups. No clustering was observed when the PCA was carried out considering as a variable the SCOBYs used. This result strengthened the theory that the kombucha aroma directly relied on the food material more than the SCOBYs used. 

**Figure 4:**
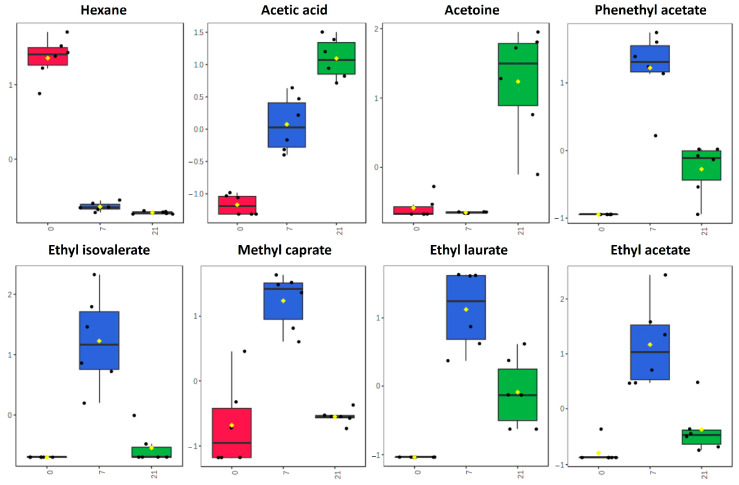
Selected marker compounds from TMEL and TAES kombucha fermented during 21 days.

To explore possible markers of kombucha fermentation, an ANOVA test was carried out. Among all the VOCs, 28 demonstrated being significant. The following VOCs were selected and studied: hexane, acetic acid, acetoine, phenethyl acetate, ethyl isovalerate, methyl caprate, ethyl laurate, and ethyl acetate (Figure 4; Figure S1). 

Among them, hexane was able to discriminate samples without fermentation from fermented ones (day 7 and day 21). Although acetic acid was detected on all days (0, 7, 21), higher levels were found with the fermentation time. Therefore, acetic acid evaluation might be enough to understand how the fermentation process is going, along with the pH value. The compound acetoin was able to distinguish samples from day 0 and 7 to day 21. This result indicated that the fermentation ending might be predicted by following this compound’s tendency. In addition, a group of different esters showed higher levels in comparison with samples that were not fermented and the end of the fermentation process in this study (day 21). An aromatic profile mainly composed of esters (fruity notes) could be attributed to a freshly fermented kombucha beverage. These compounds, especially some esters or acetic acid, could be used as potential marker compounds for fermentation control in kombuchas. 

## 4. Conclusions

Both TMEL and TAES kombuchas gave rise to interesting physicochemical, biochemical, and sensory characteristics. In general, TAES kombuchas showed lower carbohydrate and alcohol contents and the ranges of pH, viscosity, soluble proteins, and TPC were close for both truffles, with some differences, particularly at the initial days, because of the dissimilar species composition. In the case of the different SCs used, the obtained beverages differed both phyiscochemically and compositionally. As a matter of fact, kombuchas fermented with SC3 showed lower pH but higher carbohydrate content and those fermented with SC1 had lower carbohydrate and alcohol contents. With the exception of TMEL kombucha fermented with SC3, due to its high alcohol content, fermentation could be stopped within the usual bottling period (7–10 days). However, according to the results obtained, it would be recommended to extend the fermentation time up to 14 days, when lower values of carbohydrates and alcohol but higher levels of proteins and TPC were recorded, without reaching a level of acidity and viscosity that might be too high.

The obtained kombuchas showed lower sugar levels than other kombuchas (traditional and alternative) and other beverages (juices, carbonated drinks, infusions) and higher protein and phenolic contents than reported in previous studies. In addition, the kombuchas were quite aromatic compared with those obtained with fruits due to the truffle material used, although TMEL showed more VOC complexity than TAES. The kombuchas showed a high increase in acids and esters during the first 7 days, which was reflected in the pH values. Acetic acid, acetoin, or hexane compounds can be used as TAES and TMEL kombucha fermentation process control. 

These results motivate future research to evaluate the potential of truffle kombuchas, including analyses of biological activity (for instance, antioxidant and anti-inflammatory assays, due to their content of phenolic compounds) and further sensory and consumer acceptance studies. In addition, a metabolomics approach might be interesting to better understand the truffle kombucha fermentation process. 

## Figures and Tables

**Table 1:** Evolution of alcohol content (% *v*/*v*) during fermentation (21 days) in *Tuber menalosporum* (TMEL) and *Tuber aestivum* (TAES) with SCOBYs (SCs) 1, 2, and 3. Different letters denote significant differences for the same SC at different fermentation times (^A–C^) and for different SCs at the same fermentation time (^a–c^) (one-way ANOVA, Tukey’s test, *p* ≤ 0.05). n.d. = non detected.

	Alcohol Content (% *v*/*v*)						
	Day 0	Day 3	Day 7	Day 10	Day 14	Day 17	Day 21
TMEL SC1	n.d. ^A,a^	0.13 ± 0.04 ^A,b^	0.10 ± 0.04 ^A,b^	0.20 ± 0.14 ^A,b^	0.25 ± 0.21 ^A,a^	n.d. ^A,c^	n.d. ^A,a^
TMEL SC2	n.d. ^B,a^	0.65 ± 0.21 ^B,ab^	1.50 ± 0.42 ^A,a^	0.50 ± 0.14 ^B,b^	0.70 ± 0.14 ^B,a^	0.10 ± 0.00 ^B,b^	n.d. ^B,a^
TMEL SC3	n.d. ^C,a^	0.90 ± 0.14 ^B,a^	1.60 ± 0.28 ^A,a^	1.50 ± 0.14 ^AB,a^	0.40 ± 0.28 ^BC,a^	0.40 ± 0.00 ^BC,a^	n.d. ^C,a^
TAES SC1	n.d. ^B,a^	0.10 ± 0.00 ^AB,a^	0.10 ± 0.00 ^AB,b^	0.15 ± 0.07 ^A,ab^	n.d. ^B,b^	n.d. ^B,a^	n.d. ^B,a^
TAES SC2	n.d. ^B,a^	0.10 ± 0.00 ^B,a^	0.70 ± 0.14 ^A,a^	0.50 ± 0.14 ^A,a^	0.10 ± 0.00 ^B,a^	n.d. ^B,a^	n.d. ^B,a^
TAES SC3	n.d. ^B,a^	n.d. ^B,b^	0.50 ± 0.14 ^A,ab^	n.d. ^B,b^	n.d. ^B,b^	n.d. ^B,a^	n.d. ^B,a^

**Table 2:** List of volatile organic compounds identified by SPME-GC-MS in truffle kombuchas fermented using SCOBYs 1, 2, and 3 (SC1, SC2, SC3). Standard = A commercial standard was used for identification. - = No odor description.

Number	Name	CAS	RT	RI Exp	RI Nist	Odor Description *
1	Acetaldehyde	75-07-0	1.467	<500	No data	pungent, ether
2	Ethanol	64-17-5	1.568	<500	450	sweet
3	2-propanone	67-64-1	1.640	<500	500	solvent
4	Hexane	110-54-3	2.014	Standard	Standard	alkane
5	Ethyl_acetate	141-78-6	2.108	<500	612	pineapple
6	Acetic_acid	64-19-7	2.231	609	660	sour
7	Acetoine	513-86-0	3.196	717	720	butter, cream
8	3-methyl-1-butanol	123-51-3	3.470	730	730	whiskey, malt, burnt
9	2-methyl-1-butanol	137-32-6	3.506	732	733	wine, onion
10	Ethyl_isobutyrate	97-62-1	3.989	755	755	sweet, rubber
11	Isobutyruc acid	79-31-2	4.342	771	758	rancid, butter, cheese
12	2,3-butanediol	513-85-9	4.623	785	782	fruit, onion
13	Hexanal	66-25-1	5.063	803	801	grass, tallow, fat
14	Ethyl butyrate	105-54-4	5.142	805	801	apple
15	Methoxyethane	540-67-0	5.459	814	No data	-
16	Ethyl lactate	97-64-3	5.650	820	821	fruit, onion
17	Ethyl-2-methylbutyrate	7452-79-1	6.842	853	856	apple
18	Ethyl isovalerate	108-64-5	6.965	856	859	fruit
19	Isoamyl acetate	503-74-2	7.923	883	888	sweat, acid, rancid
20	2cyclopentene-1-4dione	930-60-9	8.010	885	880	-
21	Pentanoic acid	109-52-4	8.557	900	906	sweat
22	2-methyl-butanoic acid	116-53-0	8.997	912	881	cheese, sweat
23	Ethyl-2-hydroxy-isovalerate	2441-06-7	11.123	970	975	-
24	Decane	124-18-5	12.247	Standard	Standard	alkane
25	Ethyl hexanoate	123-66-0	12.311	1002	1002	apple peel, fruit
26	Hexanoic acid	142-62-1	12.500	1008	1013	sweat
27	2-ethyl-1-hexanol	104-76-7	13.248	1030	1031	rose, green
28	4-heptanol,2,6-dimehtyl	108-82-7	14.300	1061	No data	-
29	Heptanoic acid	111-14-8	15.144	1085	1085	-
30	Ethyl heptanoate	106-30-9	15.648	1100	1097	fruit
31	Undecane	1120-21-4	15.648	Standard	Standard	alkane
32	2-mehtyl-butyl-2-mehylbutyrate	2445-78-5	15.778	1104	1104	-
33	Benzene-ethanol	60-12-8	16.131	1116	1116	rose
34	Methyl octanoate	111-11-5	16.462	1126	1128	orange
35	Camphor	76-22-2	17.010	1144	1145	camphor
36	Octanoic acid	124-07-2	18.285	1184	1184	sweat, cheese
37	Ethyl octanoate	106-32-1	18.688	1197	1195	fruit, fat
38	Methyl nonanoate	1731-84-6	19.467	1224	1224	coconut
39	Ethyl phenylacetate	101-97-3	20.144	1247	1253	fruit, sweet
40	Phenethyl acetate	103-45-7	20.461	1258	1256	rose, honey, tobacco
41	Benzeneacetic acid	103-82-2	21.254	1286	1270	honey, flower
42	Ethyl nonanoate	123-29-5	21.506	1295	1293	-
43	Nonanoic acid	112-05-0	21.621	1299	1297	green, fat
44	Eicosane	112-95-8	22.284	Standard	Standard	alkane
45	Methyl caprate	110-42-9	22.350	1336	1326	wine
46	Gamma-nonalactone	104-61-0	23.358	1368	1367	coconut, peach
47	Decanoic acid	334-48-5	23.624	1374	1387	rancid, fat
48	Ethyl decanoate	110-38-3	24.151	1394	1397	grape
49	3-Methylbutyl octanoate	2035-99-6	25.498	1449	1444	-
50	2-4-ditert-butylphenol	96-76-4	27.061	1513	1518	phenolic
51	Ethyl laurate	106-33-2	29.000	1595	1595	leaf

* Odor was selected using Flavornet and The Good Scents Company’s websites. RT = retention time. RI Exp = retention index experimental. RI Nist = retention index literature database NIST.

## Data Availability

The original contributions presented in the study are included in the article/Supplementary Material, further inquiries can be directed to the corresponding authors.

## Supplementary Materials

The following supporting information can be downloaded at: https://www.mdpi.com/article/10.3390/foods13132162/s1, Table S1: List of volatile organic compounds identified by SPME-GC-MS in truffle kombuchas fermented using SCOBYs 1, 2, and 3 (SC1, SC2, SC3). In the samples name, TMEL: *Tuber melanosporum*, TAES: *Tuber aestivum*; 1, 2, and 3 correspond to the SCOBY and 0, 7 and 21 to the fermentation day; Figure S1: Selected VOCs marker compounds levels in truffle kombucha fermented during 21 days. In the sample name, TMEL: *Tuber melanosporum*, TAES: *Tuber aestivum*; 1, 2, and 3 correspond to the SCOBY.
