# Supplementary material for: Elaboration and Characterization of Novel Kombucha Drinks Based on Truffles (Tuber melanosporum and Tuber aestivum) with Interesting Aromatic and Compositional Profiles
Source: Foods. 2024 Jul 8;13(13):2162. doi: 10.3390/foods13132162 (PMC11241703; doi:10.3390/foods13132162)

Supplementary material

Table S1. List of volatile organic compounds identified by SPME-GC–MS in truffle kombuchas fermented using SCOBYs 1, 2, and 3 (SC1, SC2, SC3). In the samples name, TMEL: *Tuber melanosporum*, TAES: *Tuber aestivum*; 1,2, and 3 correspond to the SCOBY and 0, 7 and 21 to the fermentation day.

Nº	TMEL_1_0	TMEL_2_0	TMEL_3_0	TMEL_1_7	TMEL_2_7	TMEL_3_7	TMEL_1_21	TMEL_2_21	TMEL_3_21	TAES_1_0	TAES_2_0	TAES_3_0	TAES_1_7	TAES_2_7	TAES_3_7	TAES_1_21	TAES_2_21	TAES_3_21
1	0.00	0.00	0.00	0.00	0.00	0.00	0.12	0.14	0.17	0.00	0.00	0.00	0.00	0.07	0.18	0.22	0.48	0.08
2	1.60	3.07	0.82	5.34	3.51	8.09	0.99	0.68	1.31	1.04	3.16	0.59	7.91	3.82	13.46	0.00	0.48	0.21
3	5.45	4.95	6.87	0.40	0.47	0.49	0.07	0.24	0.31	16.60	7.96	5.80	0.98	0.55	0.89	0.16	0.00	0.16
4	66.51	65.74	49.69	3.55	3.81	1.63	1.03	2.35	1.25	60.04	70.00	75.72	2.86	5.30	6.61	1.01	1.80	1.98
5	0.00	4.08	0.00	10.21	11.94	16.69	4.01	3.02	10.29	0.00	0.00	0.00	24.82	10.13	18.43	1.66	3.40	1.21
6	9.16	0.00	7.98	24.35	46.33	27.79	59.17	53.28	63.53	7.32	0.00	0.00	42.55	36.47	22.47	66.33	50.56	47.89
7	0.00	0.00	3.41	0.14	0.27	0.29	12.13	16.52	4.85	0.00	0.00	1.24	0.30	0.13	0.11	22.25	20.30	20.91
8	0.97	0.74	1.23	2.21	1.47	3.21	0.28	0.08	0.40	0.63	0.51	0.00	2.64	2.11	7.78	0.00	0.00	0.00
9	4.58	2.58	5.98	0.79	0.97	1.09	0.17	0.00	0.00	0.76	0.00	0.00	0.82	0.78	1.52	0.00	0.00	0.00
10	0.45	0.66	1.48	0.24	0.24	0.31	0.04	0.00	0.00	0.00	0.00	0.00	0.07	0.14	0.43	0.00	0.00	0.11
11	2.15	0.69	3.43	0.15	0.32	0.36	1.50	0.67	0.66	0.00	0.00	0.00	0.31	0.22	0.48	0.00	0.83	0.34
12	0.00	0.00	0.00	0.18	0.45	0.72	2.84	1.50	1.53	0.00	0.00	0.00	0.17	0.83	0.28	0.00	1.35	1.47
13	0.00	0.00	0.00	0.05	0.00	0.10	0.17	0.00	0.00	0.00	0.41	0.00	0.27	0.00	0.40	0.36	1.19	1.28
14	0.00	0.00	0.00	0.15	0.12	0.22	0.10	0.00	0.00	0.00	0.00	0.00	0.00	1.55	0.00	0.00	0.00	0.00
15	0.00	0.00	0.00	0.00	0.00	0.00	0.13	0.00	0.00	0.00	0.00	0.00	0.00	0.00	0.00	0.00	0.00	0.10
16	0.00	0.00	0.00	0.55	0.50	1.54	0.00	0.42	0.32	0.00	0.00	0.00	0.86	1.08	2.25	0.00	0.00	0.19

17	0.94	0.77	1.60	0.00	0.63	0.87	0.06	0.09	0.04	0.00	0.00	0.00	0.18	0.21	0.65	0.00	0.00	0.19
18	0.00	0.00	0.00	0.33	0.24	0.38	0.00	0.04	0.11	0.00	0.00	0.00	0.22	0.14	0.46	0.00	0.00	0.00
19	0.00	0.01	0.01	0.00	0.00	0.00	0.47	0.66	0.03	0.00	0.00	0.00	0.61	0.08	0.64	0.00	6.36	0.83
20	0.00	0.00	0.00	0.00	0.00	0.00	0.00	0.00	0.00	0.00	0.00	0.00	0.00	1.59	2.10	0.00	0.00	2.80
21	0.00	0.00	0.00	2.01	4.99	5.59	3.53	2.69	3.37	0.00	0.00	0.00	0.00	2.75	0.00	1.09	0.00	0.00
22	6.41	2.87	9.95	2.21	3.05	3.63	3.01	3.04	2.80	0.00	0.00	0.00	0.00	0.93	0.00	0.41	1.63	2.77
23	0.00	0.00	0.00	0.00	0.13	0.07	0.03	0.03	0.03	0.00	0.00	0.00	0.02	0.14	0.14	0.00	0.00	0.00
24	0.09	0.13	0.00	0.00	0.00	0.00	0.00	0.00	0.00	0.09	0.00	0.06	0.00	0.00	0.00	0.00	0.13	0.00
25	0.00	0.00	0.00	0.38	0.13	0.31	0.00	0.00	0.00	0.00	0.00	0.00	0.33	0.47	1.11	0.00	0.00	0.24
26	0.00	0.00	0.00	0.00	0.53	0.00	0.23	0.36	0.17	0.00	0.00	0.00	0.73	0.79	1.21	0.92	2.08	1.30
27	0.35	0.60	0.84	0.16	0.11	0.22	0.09	0.19	0.05	0.00	0.00	0.00	0.20	0.36	0.44	0.26	0.37	0.34
28	0.00	0.00	0.00	0.11	0.07	0.15	0.07	0.06	0.03	0.00	0.00	0.00	0.10	0.25	0.38	0.00	0.16	0.19
29	0.00	0.00	0.00	0.17	0.10	0.15	0.10	0.31	0.00	0.00	0.00	0.00	0.05	0.19	0.00	0.00	0.31	0.26
30	0.00	0.00	0.00	0.88	0.33	0.47	0.01	0.00	0.00	0.00	0.00	0.00	0.12	0.41	0.85	0.00	0.00	0.00
31	0.37	0.38	0.70	0.00	0.00	0.00	0.00	0.19	0.05	0.20	0.25	0.35	0.13	0.14	0.00	0.16	0.31	0.33
32	0.10	0.00	0.00	0.13	0.00	0.00	0.00	0.00	0.00	0.00	0.00	0.00	0.00	0.00	0.18	0.00	0.00	0.00
33	0.00	0.00	0.00	0.00	0.00	0.00	0.00	0.00	0.00	0.00	0.00	0.00	0.00	0.00	0.00	0.00	0.50	0.27
34	0.00	0.00	0.00	1.57	1.78	2.29	0.68	0.36	0.37	0.00	0.00	0.00	1.53	1.88	4.95	0.47	0.48	0.46
35	0.00	0.00	0.00	0.66	0.80	0.18	0.00	0.24	0.00	0.00	1.66	0.44	0.00	1.38	0.00	0.00	0.00	0.87
36	0.15	0.15	0.38	0.00	0.00	0.00	0.00	0.00	0.00	0.03	0.00	0.15	0.00	0.00	0.00	0.00	0.00	0.00
37	0.02	2.57	0.00	1.38	1.15	1.11	0.95	1.68	1.08	1.63	0.42	1.58	1.29	1.87	0.48	0.87	1.49	2.72
38	0.00	0.00	0.00	9.41	3.68	4.26	0.57	0.69	0.41	0.64	1.39	0.20	1.86	4.11	4.68	0.60	0.49	0.41
39	0.12	0.27	0.53	1.18	0.87	0.44	0.13	0.33	0.16	0.86	4.24	1.33	0.20	1.62	0.23	0.13	0.00	2.82
40	0.00	0.00	0.00	0.13	0.14	0.21	0.18	0.13	0.22	0.00	0.00	0.00	0.06	0.06	0.15	0.00	0.00	0.07
41	0.00	0.00	0.00	0.45	0.55	0.43	0.21	0.21	0.10	0.00	0.00	0.00	0.25	0.52	0.48	0.18	0.00	0.19
42	0.00	0.00	0.00	0.00	0.00	0.00	0.00	0.00	0.00	0.00	0.00	0.00	0.00	0.00	0.00	0.00	1.69	0.56
43	0.00	0.00	0.00	25.48	8.46	13.65	1.97	2.69	1.59	3.92	3.35	2.06	2.06	8.66	2.64	0.83	0.85	2.24
44	0.00	9.47	4.08	2.79	0.00	0.61	3.86	5.68	3.99	4.92	5.43	9.28	3.95	5.40	0.87	0.82	0.84	2.06
45	0.00	0.00	0.00	0.09	0.19	0.12	0.00	0.16	0.00	0.00	0.31	0.22	0.07	0.21	0.08	0.00	0.00	0.42

46	0.00	0.00	0.00	0.14	0.09	0.17	0.15	0.19	0.17	0.00	0.00	0.00	0.10	0.21	0.20	0.20	0.43	0.57
47	0.00	0.00	0.00	0.52	0.32	0.66	0.41	0.54	0.26	0.00	0.00	0.00	0.19	0.47	0.00	0.28	0.63	0.46
48	0.00	0.00	0.00	1.28	0.92	1.16	0.32	0.33	0.24	0.77	0.43	0.25	0.83	1.21	1.23	0.40	0.31	0.32
49	0.00	0.00	0.00	0.08	0.13	0.16	0.06	0.04	0.02	0.00	0.00	0.00	0.00	0.12	0.08	0.04	0.00	0.00
50	0.56	0.27	0.73	0.07	0.06	0.08	0.10	0.10	0.07	0.51	0.47	0.71	0.25	0.53	0.39	0.31	0.40	0.30
51	0.00	0.00	0.00	0.06	0.08	0.07	0.02	0.04	0.02	0.00	0.00	0.00	0.11	0.11	0.11	0.04	0.07	0.06

Figure S1. Selected VOCs marker compounds levels in truffle kombucha fermented during 21 days. In the samples name, TMEL: *Tuber melanosporum*, TAES: *Tuber aestivum*; 1,2, and 3 correspond to the SCOBY.

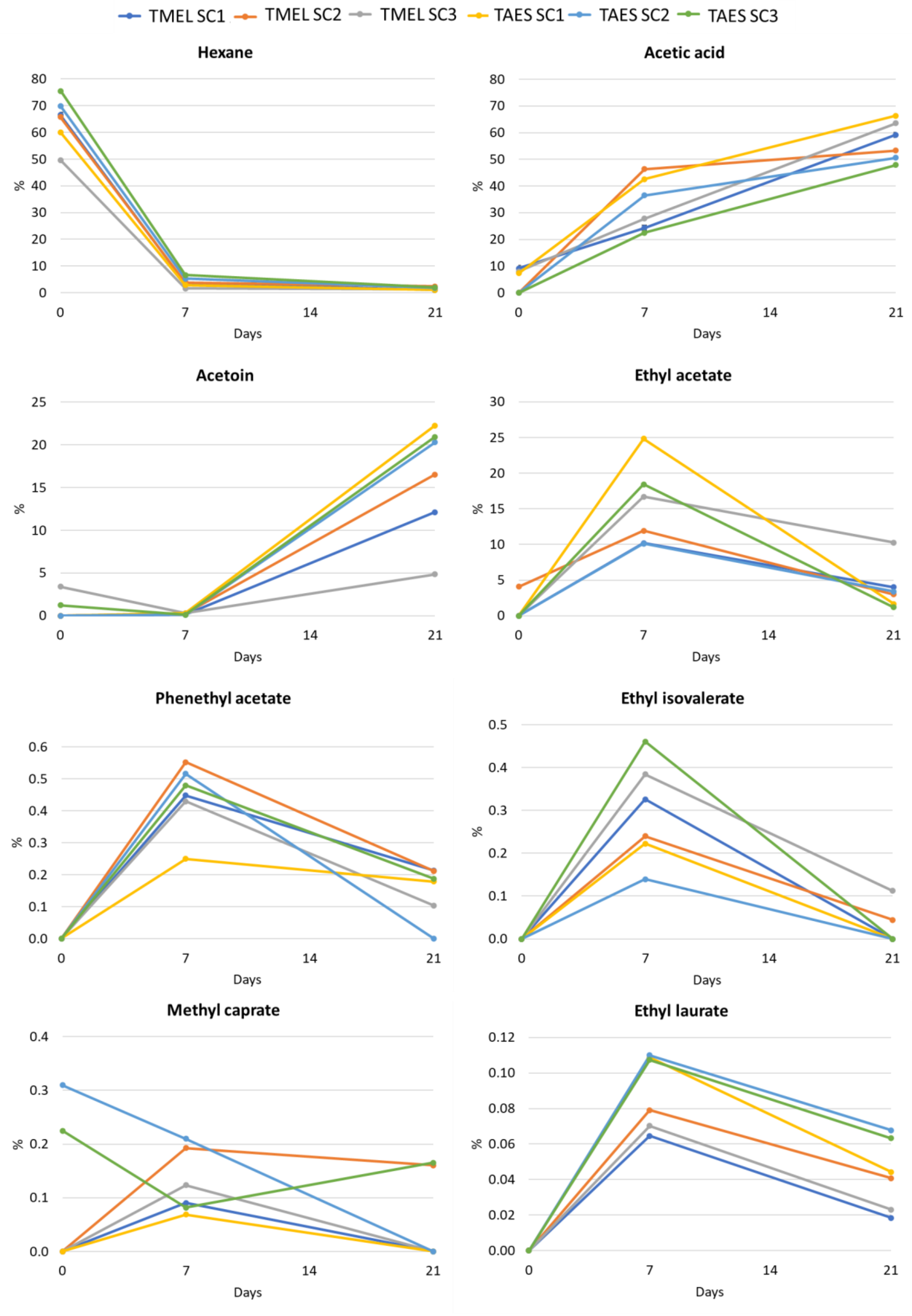

Supplement: Supplementary file 1 [file foods-13-02162-s001.zip › foods-3081538-supplementary.pdf]
